# The effect of cognitive-behavioral therapy and haptonomy on fear of childbirth in primigravida women: a randomized clinical trial

**DOI:** 10.1186/s12888-023-05414-3

**Published:** 2023-12-11

**Authors:** Zahra Alivand, Roghaiyeh Nourizadeh, Sevil Hakimi, Khalil Esmaeilpour, Esmat Mehrabi

**Affiliations:** 1 Student Research Committee, Midwifery Department, Tabriz University of Medical sciences, Tabriz, Iran; 2 Midwifery Department, Faculty of Nursing and Midwifery, Tabriz University of Medical sciences, Tabriz, Iran; 3 Faculty of Education and Psychology, University of Tabriz, Tabriz, Iran

**Keywords:** Fear, Birth, Methods, Cognitive-behavioral therapy, Maternal-fetal relations

## Abstract

**Background:**

Considering the role of fear of childbirth (FOC) in the enhancement of unnecessary cesarean sections (CS), the present study aimed at evaluating the effect of Cognitive-Behavioral Therapy (CBT) and haptonomy on the FOC (as primary outcome) and intended birth method and final birth method (as secondary outcomes) among primigravida women.

**Methods:**

This randomized clinical trial was conducted on 99 primigravida women in Tabriz, Iran 2022. Participants were assigned to three groups with a ratio of 1:1:1 using stratified block randomization based on the fear intensity. One of the intervention groups (n = 33) received eight group sessions of CBT from 24 to 28 weeks of gestation and the other intervention group (n = 33) received haptonomy during seven sessions once a week. The control group (n = 33) received routine prenatal care. The Wijma questionnaire was completed by the participants before the intervention, after the intervention at 35–37 weeks of gestation, and after birth. The intended birth method was investigated before and after the intervention at 35–37 weeks of gestation. The final birth method and the reasons for CS were recorded based on the mother’s medical profile. The one-way ANOVA was used before the intervention and RMANOVA after the intervention to compare the mean scores of FOC among the three groups. Further, chi-square test was applied to compare the intended and final birth method.

**Results:**

The mean (standard deviation: SD) of FOC in the CBT group changed from 74.09 (11.35) at 24–28 weeks of gestation to 46.50 (18.28) at 35–37 weeks and 48.78 (20.64) after birth (*P* < 0.001). The means (SDs) of FOC in the haptonomy group were 76.81 (13.09), 46.59 (15.81), and 45.09 (20.11), respectively (*P* < 0.001). The mean (SD) of FOC in the control group decreased from 70.31 (6.71) to 66.56 (18.92) and then, increased to 71.00 (21.14) after birth (*P* = 0.878). After the intervention, there was no statistically significant difference among the three groups in terms of the intended birth method (*P* = 0.278), and final birth method (*P* = 0.107).

**Conclusion:**

The findings of the present study revealed that both CBT and haptonomy interventions reduce FOC. Although the desire for vaginal birth and final vaginal birth in the haptonomy group was more than that in the other two groups, there was no statistically significant difference among the three groups.

**Trial registration:**

Iranian Registry of Clinical Trials: IRCT20170506033834N9. Date of registration: 02.01.2022. URL: http://en.irct.ir.

## Background

Pregnancy is considered as a very important and short-term experience with long-term effects on women’s life [1]. This period is unknown, especially for women experiencing their first pregnancy, as being in an unknown situation can lead to fear and worry [2]. Most of women, especially primigravida women, experience a reasonable fear, since they are unfamiliar with the childbirth process [3]. Some factors, such as fear of pain, death, and unexpected problems, poor self-efficacy, and worry about baby’s health are regarded as the main reasons for FOC [4].

The global prevalence of severe FOC was reported 16% among low risk pregnant women [5]. The prevalence of FOC among Iranian pregnant women is reported to be 59% [6] and about 6–13% of them experience severe and debilitating fear [7]. Mother’s anxiety and FOC are associated with consequences, such as premature birth, low birth weight baby, intrauterine fetal growth restriction, abnormal fetal heart rate patterns, low Apgar of the baby, and increased perinatal morbidity [8, 9]. Hypoxia caused by the decrease in blood flow to the pelvic muscles in response to the enhancement of catecholamines and serum cortisol, due to fear, can increase mothers’ pain in labor [10].

FOC is acknowledged as one of the important reasons to request elective CS among primigravida women, leading to the repeated CS following the initial CS [11]. Over recent years, global CS rate has significantly increased from around 7% in 1990 to 21%, surpassing the acceptable CS rate which is about 10–15% raised by the WHO [12]. These trends are expected to reach the global rate of 29% by 2030 [13]. Although the complications of CS are more than vaginal birth, about 70% of CS are performed without a medical indication in Iran [14].

Some of the complications of CS compared to vaginal birth include anesthesia complications, post-operative infection, more bleeding, and risk of embolism [15]. The severe FOC can even lead to the abortion and avoidance of consequent pregnancies [16].

In literature review, different interventions, such as group psychotherapy [17], relaxation [18], music therapy [19], massage therapy [20], aromatherapy [21], individual counseling by midwives [22], childbirth preparation classes [23, 24], haptonomy [25], CBT [26], and mindfulness-based counseling [27] have been used to reduce FOC. One of the relatively effective methods to reduce FOC is CBT-based counseling approach, helping clients to develop skills to improve their cognitive reconstruction [26]. This counseling method is a combination of cognitive and behavioral approaches, aiming to identify and challenge irrational behaviors and thoughts [28, 29]. A systematic review and meta-analysis study indicated that CBT is effective in reducing FOC, especially in Middle Eastern countries [26].

Another relatively new method for reducing FOC is haptonomy intervention, including communication techniques between the mother’s mind and body and raising mother’s self-awareness about her ability for the physiological process of pregnancy and childbirth, strengthening feelings and a positive attitude towards pregnancy, and the fetal-mother communication techniques, such as touch and interaction with the fetus [30–32]. According to the early studies, this technique has a significant effect on reducing FOC [25, 30, 33].

In literature review, both CBT and haptonomy interventions are identified to be effective in reducing FOC [25, 26, 30, 33]. However, to the best of our knowledge, no study was found to compare educational and psychological interventions to identify the most effective methods to reduce FOC. Considering the importance of education and counseling among primigravida women in preventing elective CS [34], the need for preventive and interventional measures in this area, and due to the lack of the comparison of the effect of educational and psychological interventions on FOC, the present study aimed at comparing the effect of CBT and haptonomy interventions on FOC among primigravida women.

### Study hypotheses

1. The mean score of FOC differs between CBT, haptonomy, and control groups.
2. Intended birth method and final birth method differs between these three groups.

## Method

### Study design and participants

This study is the result of a research project approved by Vice Chancellor for Research of Tabriz University of Medical Sciences, Faculty of Nursing and Midwifery with ethics code IR.TBZMED.REC.1400.678 and registration code of (IRCT20170506033834N9) on Iranian Registry of Clinical Trials site. This randomized controlled clinical trial was carried out on 99 pregnant women referred to health centers in Tabriz, Iran from January 5 to August 11, 2022.

The inclusion criteria were primigravida women aged 18–45 years with gestational age of 24–28 weeks and a Wijma Delivery Expectancy/ Experience Questionnaire (WDE-Q) score above 65 [35]. The exclusion criteria included having a history of mental disorders before and during pregnancy based on the health records, high risk pregnancies, such as hypertension, diabetes, placenta previa, cardiopulmonary diseases, and other chronic diseases, intermittent bleeding during pregnancy, incompetent cervix, multiple pregnancy, unplanned pregnancy, history of recurrent miscarriages, and pregnancy with assisted reproductive technologies, and having an indication for CS.

The sample size was calculated based on the FOC variable in the study of Dorosti et al. [33] using G Power software. Considering m_1_ = 93, m_2_ = 79.05, with the assumption of 15% reduction, SD1 = SD2 = 16.93, and two-sided hypothesis, and Power = 90%, sample size was obtained 30 in each group and regarding 10% attrition, the final sample size was 33 in each group.

### Sampling

Sampling was done in eleven crowded health centers with different socio-economic levels in the city. The researcher attended the selected health centers and after preparing a list of primigravida women aged 18–45 years with gestational age of 24–28 weeks, called and evaluated them in terms of the inclusion and exclusion criteria and invited the eligible women. Participants completed WDE-Q in the face-to-face session and the objectives and method of the study were explained for women who gained WDE-Q score above 65. The eligible women completed the written informed consent form to participate in the study and the demographic and obstetric form. Then, their intended birth method was recorded.

### Randomization

Participants were assigned to three groups with a ratio of 1:1:1 using stratified block randomization based on the high (66–85) and severe (> 85) FOC [35] using Random Allocation Software (RAS) with a block size of 9. Stratified randomization prevents intergroup imbalance for known factors, influencing experimental responsiveness. As a result, stratification may prevent type 1 error and improve power for small trials [36]. Central randomization by phone was used as the allocation concealment method. Further, participants were not blinded, due to the nature of the interventions. However, the outcome assessor was blinded. Blinding of outcome assessor reduces detection bias [37].

### Intervention

Eight group sessions of CBT were held for one of the intervention groups from 24 to 28 weeks of gestation for 45–60 min once a week. Counseling sessions were held by a clinical psychologist. Table 1 indicates the content of counseling based on the intervention protocol [28].

**Table 1:** The content of cognitive behavioral therapy and haptonomy sessions for fear of childbirth in primigravid women

session	Content of haptonomy intervention	Content of cognitive behavioral therapy intervention
First	Expressing the goals and logic of haptonomy, explaining the process and stages of pregnancy and childbirth in line with the nature ability of woman for pregnancy and childbirth through the explanation of physiological, anatomical, and hormonal changes during pregnancy and showing the video of the effect of pregnancy hormones of labor on the anatomical changes of the pelvis	Stating the objectives and logic of cognitive-behavioral therapy, increasing their knowledge and awareness about the process of pregnancy and childbirth
Second	Encouraging women to express their worries and stressful issues regarding pregnancy and childbirth and helping mothers to solve and replace their fears and worries **Assignment**: Recording the perceived threats and concerns regarding childbirth in one coloumn and recording positive feelings and attitudes toward pregnancy in the other coloumn of paper	Expressing the importance of fear of childbirth, investigating its causes and its impact on the process of pregnancy and childbirth, and helping mothers to identify the causes of their fear **Assignment**: Writing their expected changes from the treatment/therapy plan as assignment for the next session
Third	Training different stages of fetal growth and development	Identifying ineffective and irrational thoughts of a person about her ability to give birth, examining the attitudes of women with FOC and their dominant negative attitudes, examining illogical beliefs and explaining them, and introducing cognitive distortions **Assignment**: Iidentifying the cases of cognitive distortions and ineffective beliefs and revise cognitive distortions
Fourth	Training communication methods, such as talking to the fetus, observing and touching the abdomen, paying attention to the movements of the fetus in response to the mother’s voice and touching or shaking the abdomen (playing with the fetus) and recommending calling the fetus by name **Assignment**: Counting and recording the fetal movements on a sheet and bring it in the next session	Resolving misunderstandings caused by wrong perceptions (cognitive skills), and training how to increase positive self-talks and problem-solving skills and their role in reducing FOC
Fifth	Increasing the mother’s awareness about the mind-body connection and strengthening feelings and positive attitude toward pregnancy through training various methods of labor pain control and breathing techniques. **Assignment**: Practicing breathing techniques at home and requesting to record the birth plan designed by the mother on paper and bring it in the next session.	Introducing the ABC cycle (activating events, beliefs, and consequences), stating the advantages and disadvantages of vaginal birth and CS, training the stages of childbirth **Assignment**: Practicing coping styles with and preventing inappropriate behaviors and thoughts
Six	Training the effect of changing the mother’s position during labor on the pelvis on the moulage and training how to control anxiety and negative thoughts	Training distraction techniques, training and practicing relaxation, breathing techniques and Kegel exercises **Assignment**: Practicing relaxation
Seventh	Practicing breathing techniques, examining the fetus’s reactions to the mother’s touch and calling, and summarizing the contents.	Searching for common mental images of women during pregnancy, training the technique of changing mental images **Assignment**: Practicing how to cope with the previous attitudes when they come again
Eighth	-	Summarizing the contents and evaluating the different techniques trained, giving feedback about the effectiveness or ineffectiveness of counseling

FOC: fear of childbirth; CS: cesarean section

Another intervention group, received haptonomy in groups of 3 people during seven sessions from 24 to 28 weeks of gestation for 45–60 min once a week. A significant and positive correlation was found between the WDE-Q mean scores of the pregnant women and gestaional age [38]. Given that the FOC increases by approaching the delivery, 24–28 weeks of gestation were chosen for interventions. All haptonomy sessions were held by the author, who had an international certificate for haptonomy intervention. Table 1 demonstrates the content of the sessions according to the intervention protocol [25].

The researcher sent the intervention groups reminder messages one day before each session to increase the participants adherence to the interventions. All interventions were presented in the counseling room of the nearest health center to the participants’ home. The control group received routine prenatal care. During the gestational age of 35–37 weeks, the researcher called the participants and asked them to fill out the WDE-Q when they attended the health centers for antenatal care. In the following, their intended birth method was asked and indications for CS were evaluated. Participants completed WDE-Q again after childbirth, and birth method was recorded according to the mother’s medical profile.

### Data collection tools

#### Demographic and obstetric characteristics form

This form included the variables of age, education, occupation, family income, history of abortion/ ectopic pregnancy, and gender of the fetus which were completed by the participants before the intervention.

### Wijma delivery expectancy/ experience questionnaire (WDE-Q)

The WDE-Q was employed to evaluate FOC. Mothers specified their feelings based on a 6-point Likert scale ranging from 0 to 5. The total score range is between 0 and 165, as higher score indicates more FOC, a score of 66–85 represents high FOC, and a score above 85 denotes severe FOC. The Cronbach’s alpha coefficient and Intra Class Correlation Coefficient (ICC) of the instrument were reported 0.70 and > 0.9, respectively [35]. The validity and reliability of WDE-Q have been verified by Mortazavi in Iran and its Cronbach’s alpha coefficient has been reported as 0.914 [39]. This questionnaire was completed by the participants at 24–28 weeks, 35–37 weeks, and after giving birth.

### Birth method checklist

The question of “If there is no medical prohibition for vaginal birth, which birth method do you prefer?“ was asked before the intervention at 24–28 weeks of gestation and after intervention at 35–37 weeks to know women’s preferred birth method. Birth method was recorded according to the mother’s medical profile. In the case of CS, the reasons were recorded.

### Data analysis

The collected data were analyzed by SPSS_24_ software and Shapiro-Wilk test was used to assess the data normality. Descriptive statistics as mean (SD), and frequency (%) were used in the analysis of data collected in this study. Chi-Square test and One-way ANOVA were used to compare the socio-demographic characteristics of three groups. The One-way ANOVA was employed before the intervention and RMANOVA after the intervention to compare the mean scores of FOC among the three groups. Furthermore, chi-square test was applied to compare the intended and final birth method. The intention to treat (ITT) method was employed for data analysis, which means “once randomized and always analyzed, regardless of noncompliance and protocol deviations’’ [40]. The p-values below 0.05 were considered statistically significant.

## Results

**Fig. 1:**
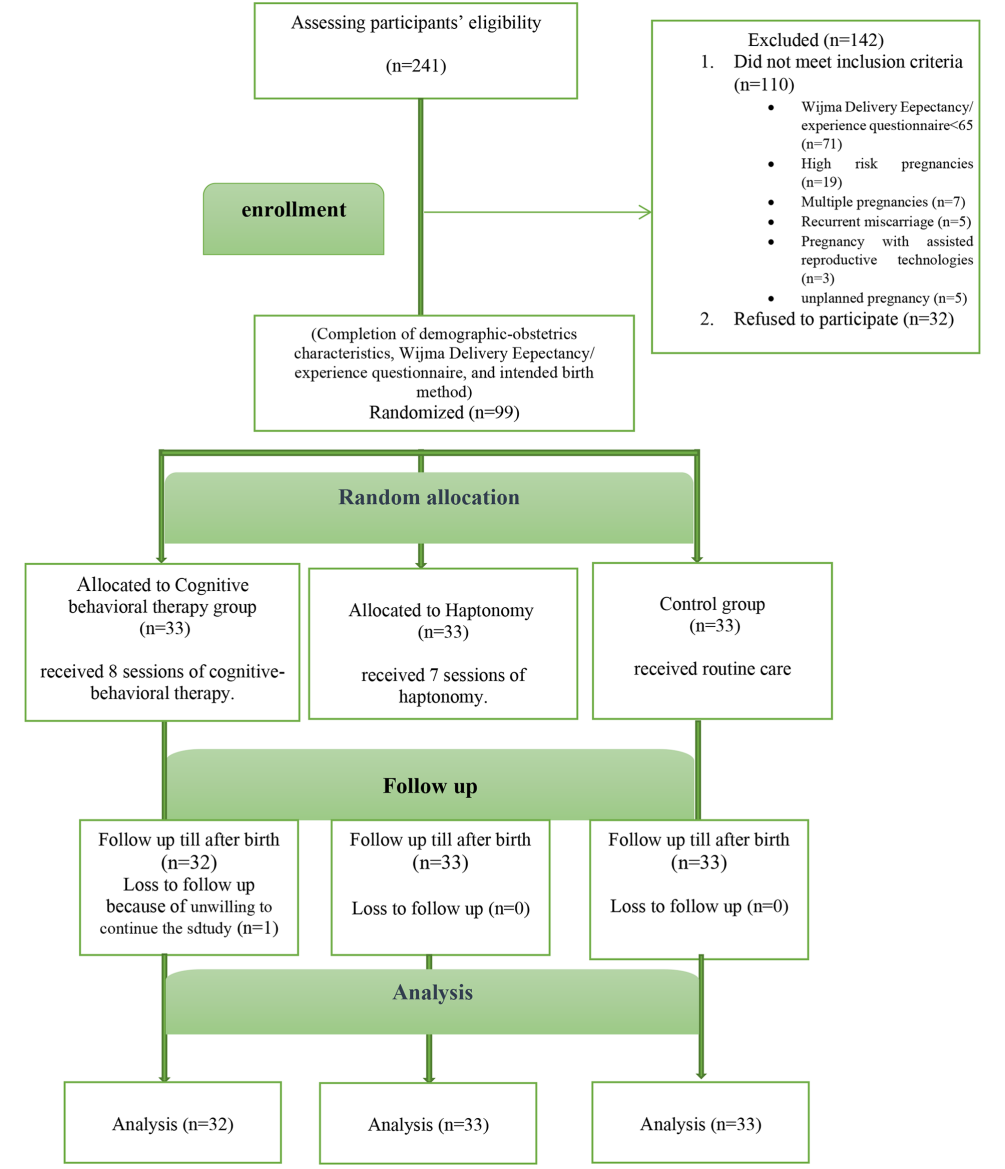
Enrollment of participants in the study

A total of 241 pregnant women were examined, of whom 110 women were excluded, due to not fulfilling eligibility criteria. Eventually, 99 individuals participated in the study and were randomly assigned to the CBT, haptonomy, and control groups. In the CBT group, one person was reluctant to continue the study (Fig. 1). There was no statistically significant difference among the groups in terms of the demographic and obstetric characteristics (Table 2).

**Table 2:** The characteristics of primigravid women in the CBT, haptonomy, and control groups

Variable	CBT^†^ group (n = 32) N (%)	Haptonomy group (n = 33) N (%)	Control group (n = 33) N (%)	P
**Age**‡	25.82 (6.12)	25.15 (5.19)	23.27 (5.19)	0.158*
**Level of education**				
Secondary school	4 (12.5)	6 (18.2)	11 (33.3)	0.312**
High school / diploma	15 (45.8)	18 (54.5)	13 (39.4)	
Associate’s degree / Bachelor’s degree	8 (25.0)	6 (18.2)	7 (21.2)	
Master’s degree / PhD	5 (15.6)	3 (9.1)	2 (6.1)	
**Occupation**				
Housekeeper	27 (84.3)	30 (90.9)	32 (97.0)	0.142**
Employed	5 (15.6)	3 (9.1)	1 (3.0)	
**Monthly income( $US)**				
< 200	13 (40.6)	11 (33.3)	12 (36.4)	0.672**
200–500	17 (53.1)	16 (48.5)	17 (51.5)	
> 500	2 (6.2)	6 (18.2)	4 (12.1)	
A history of one abortion / ectopic pregnancy	4 (12.5)	2 (6.1)	5 (15.2)	0.614**
**Gender of the fetus**				
Female	15 (46.8)	16 (48.5)	19 (57.6)	0.672**
Male	17 (53.1)	17 (51.5)	14 (42.4)	

†Cognitive behavioral therapy, ‡Mean (SD), *One-way ANOVA, **Chi-Square test,

**Fig. 2:**
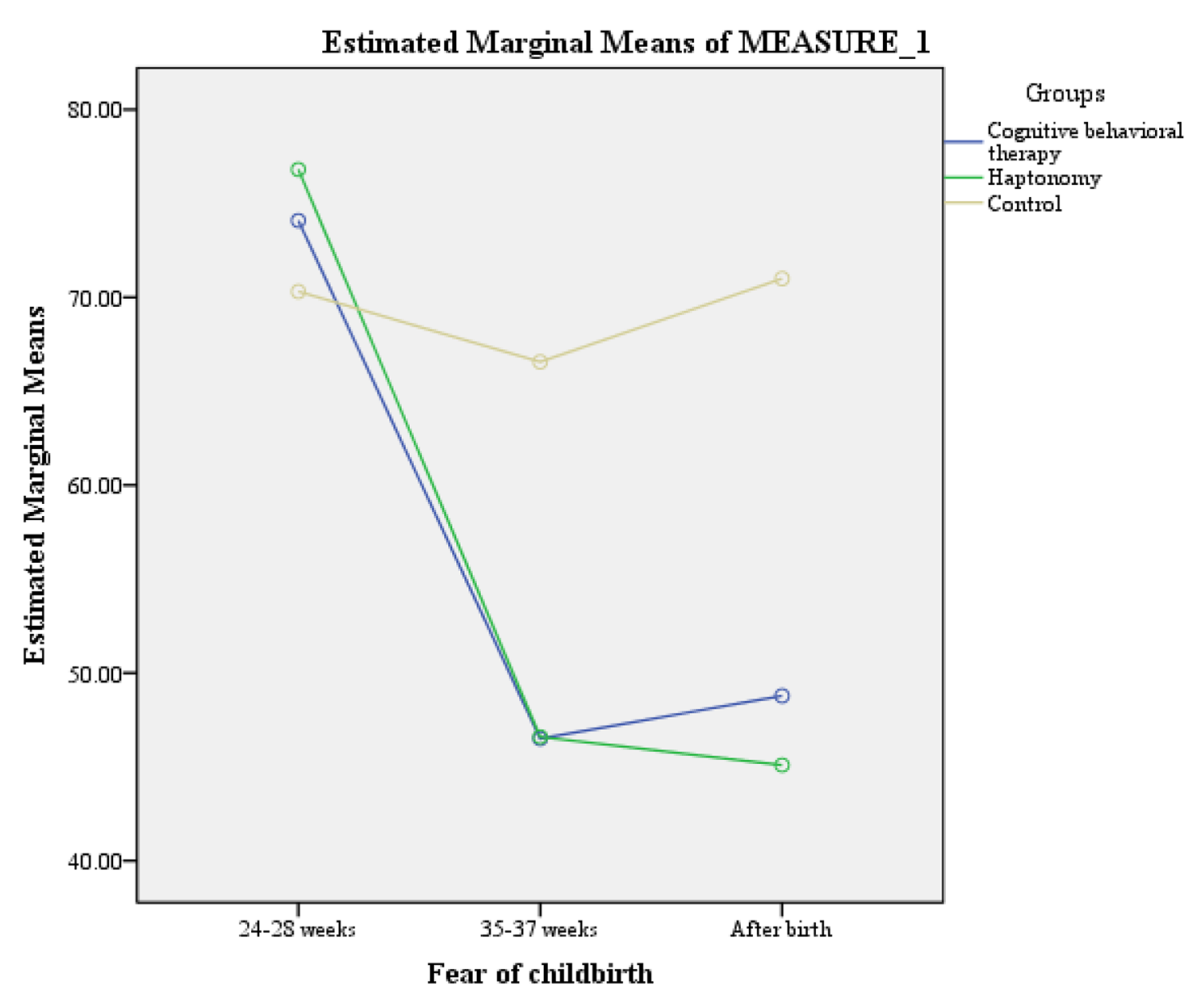
The comparison of fear of childbirth among cognitive behavioral therapy, haptonomy, and control groups

After the intervention, a statistically significant difference was observed in the FOC score among the three groups (*P* < 0.001) (Table 3). So that, the trend of FOC in the control group was different from that in the intervention groups (Fig. 2). Following the intervention, no statistically significant difference was found in terms of FOC among the haptonomy and CBT groups at 35–37 weeks [Mean difference (MD): -0.09 ( 95%CI: -8.89 to 8.70), *P* = 0.983], and after birth [MD: 0.29 (95%CI: -7.25 to 7.84, *P* = 0.891].

**Table 3:** The comparison of the mean score of primigravid women´s fear of childbirth in the CBT, haptonomy, and control groups during the study

Fear of childbirth	CBT† (n = 32) Mean (SD)	Haptonomy (n = 33) Mean (SD)	Control (n = 33) Mean (SD)	P
Before intervention	74.09 (11.35)	76.81 (13.09)	70.31 (6.71)	0.034*
35–37 weeks	46.50 (18.28)	46.59 (15.81)	66.56 (18.92)	< 0.001**
After birth	48.78 (20.64)	45.09 (20.11)	71.00 (21.14)	< 0.001***
P*	< 0.001	< 0.001	0.878	-

† Cognitive behavioral therapy, *One-way ANOVA, **ANCOVA after adjusting the baseline value, ***RMANOVA after adjusting the baseline value and birth method

Although after the intervention, the desire for vaginal birth in the haptonomy group was more than that in the other two groups at 35–37 weeks, there was no statistically significant difference among the three groups in terms of intended birth method (*P* = 0.278). In the comparison of the final birth method among the three groups, although vaginal birth in the haptonomy group was more than that in the CBT and control groups, the difference was not statistically significant (*P* = 0.107) (Table 4). Based on the birth record, the elective CS request in the CBT, haptonomy, and control groups was 12 (37.5), 12 (36.4), and 18 (54.5), respectively. CS with medical indication in the CBT group 11 (34.3) was higher than that in the haptonomy 6 (18.1) and control groups 8 (24.2) (*P* = 0.143).

**Table 4:** The comparison of the intended and final birth method in the CBT, haptonomy, and control groups before and after the intervention

Variable		CBT† group (n = 32) N (%)	Haptonomy group (n = 33) N (%)	Control group (n = 33) N (%)	P*
Intended birth method at 24–28 weeks	Vaginal	9 (28.1)	13 (39.4)	14 (42.4)	0.654
	CS‡	23 (71.8)	20 (60.6)	19 (57.6)	
Intended birth method at 35–37 weeks	Vaginal	11 (34.4)	17 (51.5)	11 (33.3)	0.278
	CS	21 (65.5)	16 (48.4)	22 (66.6)	
Final birth method	Vaginal	9 (28.1)	15 (45.5)	7 (21.2)	0.107
	CS	23 (71.8)	18 (54.5)	26 (78.7)	

†Cognitive behavioral therapy, ‡Cesarean section, *Chi-square test

## Discussion

The present study for the first time compared the effect of CBT and haptonomy interventions on FOC. Based on the results of the present study, CBT and haptonomy interventions significantly reduced FOC at 35–37 weeks of gestation and after birth compared to the control group. However, there was no significant difference between the intervention groups. In line with the findings of the present study, Ucar and Golbasi [41] investigated primigravida women at 20–32 weeks of gestation and indicated that FOC in the intervention group significantly reduced compared to the control group after 6-session of CBT, including recording nonfunctional thoughts, teaching the ABC model (activating events, beliefs, and consequences) and relaxation techniques, and creating a positive perception of birth to enable pregnant women to cope with their FOC.

In another study, Ghazaei et al. [42] revealed that 9-session of CBT were more effective in reducing FOC of primigravida pregnant women at 16–24 weeks of gestation compared to the psycho-education group. The results of a systematic review demonstrated that CBT is effective in the FOC reduction [26]. Further, Shahsavan et al. [43] examined 102 primigravida women with severe FOC at 30 weeks of gestation and found that internet-based cognitive-behavioral therapies significantly reduced FOC in the intervention group compared to the control group. However, Tata et al. [44] reported no statistically significant difference in FOC of primigravida women between the intervention and control groups at 36 weeks of gestation, following 6-session of Beck’s group cognitive therapy, including explaining about the delivery process and labor phases, fear of childbirth and its physical and psychological consequences and the relationship between FOC and anxiety, cognitive-behavioral techniques, such as relaxation, Socratic dialogue, and thoughts. The difference between the results of the aforementioned and present study can be attributed to the difference in the content of the intervention and the number of sessions, as the number of sessions in the study of Tata et al. was fewer than that in our study. In addition to the cognitive strategies used in the study of Tata et al. [44], the present study employed other cognitive strategies, such as visualization techniques (producing mental images incompatible with pain) and positive self-talk (emphasizing one’s ability to bear the pain of childbirth), and behavioral techniques, including distraction, relaxation, breathing techniques, and Kegel exercises.

In the present study, FOC in the haptonomy group was less than that in the other two groups after intervention. Consistent with the findings of the present study, in the study of Ozbek and pinar [25], following 7-sessions of haptonomy intervention on pregnant women at gestational age of 22–28 weeks, the mean score of FOC in the haptonomy group significantly decreased compared to the control group. In the study of Adam [45], FOC decreased due to the increase in women’s self-confidence following haptonomy intervention. In addition, Klabbers et al. [30] reported a significant reduction in the FOC mean score of pregnant following 8-session of haptonomy. In the study of Dorosti [33] on primigravida women with high and severe FOC, a significant reduction in the FOC was observed in the intervention group compared to the control group following five sessions of haptonomy intervention. To the best of our knowledge, the results of all studies investigated the effect of haptonomy on FOC are consistent and this has been acknowleged as an effective technique in reducing FOC.

In the present study, although the desire for vaginal birth and final vaginal birth in the haptonomy group was more than that in the other two groups and the request for elective CS was less in the haptonomy and CBT groups, there was no statistically significant difference in terms of final birth method, and the rate of CS among the three groups. In this study, the rate of caesarean section with medical indication in the CBT group was higher than that in the other two groups. It is worth mentioning that choosing the birth method is not just a personal matter and several factors, such as medical indications, spouse preference, and socio-cultural factors are involved in this regard [46, 47].

In line with the results of the present study, Tata et al. [44] indicated no significant difference between the intervention and control groups in terms of the frequency of vaginal birth following 6-session of Beck’s cognitive therapy. However, the results of a study reported that group consultation using cognitive technique is an appropriate approach to improve knowledge, attitudes, and tendencies of mothers toward vaginal birth [48]. Inconsistent with the findings of the present study, in the study of Ghazaie et al. [34] on primigravida women preferred CS due to the fear of vaginal birth before the intervention, a significant difference was reported in terms of the intended and final CS between the intervention and routine care groups after 9 sessions of CBT. In another study, Shahsavan et al. [43] following eight sessions of internet-based cognitive-behavioral therapy for primigravida women with severe FOC reported that the intervention significantly reduced the elective CS.

### Strength and limitations

The present study was the first one evaluated the effect of haptonomy intervention on the birth method and no study was found in the literature review in this area. One of the limitations of the present study was the impossibility of blinding of participants due to the nature of study. In addition, this study was conducted on primigravida women, and the results cannot be generalized to multiparous women with secondary FOC caused by traumatic childbirth experience. Duration of laber and social support were not measured in this study which could affect the FOC score after birth.

## Conclusion

Based on the findings of the present study, CBT and haptonomy reduce the FOC. Given that the desire for vaginal birth and final vaginal birth in the haptonomy group was more than that in the other two groups, midwives are suggested to use haptonomy intervention as a midwifery skill and preferred approach in reducing FOC, due to the lack of need for psychological techniques of CBT. It is suggested to integrate haptonomy intervention in the care package of primigravida women with FOC. It is recommoned to perform more studies regarding the impact of haptonomy on the birth method. Also, it is suggested to conduct a similar study on multiparous women with secondary FOC to compare the results.

## Data Availability

The datasets used and / or analyzed during the current study are available from the corresponding author on reasonable request.

## Abbreviations

FOC: Fear of Childbirth
CBT: Cognitive-Behavioral Therapy
SD: Standard Deviation
IRCT: Iranian Registry of Clinical Trials
RAS: Random Allocation Software
CI: Confidence Interval
CS: Cesarean Section
WDE-Q: Wijma Delivery Expectancy/ Experience Questionnaire
